# Duration of oestrogen exposure does not affect reproductive outcome in artificial cycles: a retrospective analysis of more than 7000 hormonal replacement therapy cycles for an embryo transfer

**DOI:** 10.3389/fendo.2023.1233685

**Published:** 2023-08-01

**Authors:** Cristina Rodríguez-Varela, Maria Salvaleda-Mateu, Elena Labarta

**Affiliations:** ^1^ Valencian Infertility Institute (IVI) Foundation – Health Research Institute (IIS) La Fe, Valencia, Spain; ^2^ Human Reproduction Department, Valencian Infertility Institute (IVI) Reproductive Medicine Associates (RMA) Valencia, Valencia, Spain

**Keywords:** oestrogen, artificial cycle, pregnancy, embryo transfer, *in vitro* fertilization

## Abstract

**Introduction:**

Optimal duration of oestrogen exposure before an embryo transfer in artificial cycles has not been defined yet, as its correlation with reproductive outcome remains controversial. The length of oestrogen treatment before starting luteal phase support varies significantly among patients.

**Materials and methods:**

In this study, we conducted a retrospective analysis of a huge database of our own clinical results in artificial cycles in the past five years. The aim of this study was to assess the effect of the length of estrogen exposure on reproductive outcome and to evaluate if there is any optimal duration of estrogen exposure in order to maximize success rates.

**Results:**

Differences in pregnancy rates according to oestrogen length, if present, were not clinically relevant.

**Discussion:**

Our results suggest that the length of oestrogen exposure (in days) before exogenous progesterone administration do not affect clinical outcomes.

## 1 Introduction

Artificial endometrial preparation with hormonal replacement therapy (HRT) is frequently used for frozen embryo transfer (FET) and egg donation cycles (1). This protocol involves the administration of exogenous estrogen and progesterone trying to mimic the hormonal changes happening physiologically in a natural cycle.

Whereas the number of days of progesterone administration until the embryo transfer (ET) is clearly defined according to the embryo development stage, the part of the protocol that involves the length of the oestrogen exposure is highly variable among patients. The latter usually extends between six and twenty-five days before progesterone administration onset, although it may vary relying on the ability of each patient to reach the minimum endometrial thickness, as well as special conditions such as personal decisions or waiting for the donor to be ready in oocyte donation cycles. However, the optimal duration of oestrogen exposure in HRT cycles for an ET hasn’t been defined yet, and its correlation with reproductive outcome remains controversial.

Several studies have claimed that the duration of oestrogen exposure before ET does not affect the final pregnancy outcome in cycles with own oocytes, both with (2) or without preimplantation genetic testing for aneuploidies (PGT-A) (3). These two above mentioned studies covered a range of the oestrogen exposure therapy between 10 and 39 days, approximately. In contrast, Bourdon et al. claimed that a duration of oestrogen exposure until ET longer than 29 days was correlated to significantly lower live birth rates (LBR), and a length longer than 36 days was correlated to significantly higher probability of miscarriage (4).

While these studies evaluated the impact of the length of oestrogen exposure until the day of ET (including the luteal phase prior to transfer), the vast majority of studies addressing this issue have analyzed their results by comparing the length of oestrogen exposure until the day of onset of progesterone administration. In this regard, Jiang et al. in (5) did not find any significant effect on pregnancy rates between 7 or 14 days of oestrogen exposure (5). Indeed, higher clinical pregnancy rates were observed in a group of patients without pituitary suppression and a length of oestrogen exposure shorter than 20 days, in comparison to a length of 20 days or more (6).

In addition, in oocyte donation cycles, the length of estrogen exposure can be also extended in order to facilitate the synchronization between patient and donor. In this type of cycles, the optimal length of oestrogen exposure seems to be between 12 and 19 days, approximately (7, 8).

Nevertheless, the length of oestrogen exposure may exert an effect in other parameters, such as gestational age at delivery. In Sekhon et al. (2), in spite of having denied the correlation between the length of oestrogen exposure and pregnancy rates, each additional day of estrogen therapy was significantly associated with a reduction in the gestational age at delivery (in weeks) (β = -0.07 ± 0.03, p = 0.01) (2).

Finally, the onset of oestrogen therapy (day 2-5 of cycle vs. day 6 onwards), and not its length, has been proven to have a significant impact on endometrial thickness, as well as on biochemical and clinical pregnancy, being higher in the late onset group. In contrast, the same relationship has not been proven for ongoing pregnancy rate (9).

The aim of the current study is to retrospectively analyze the impact of the duration of oestrogen exposure (in days) until the onset of progesterone administration on pregnancy outcome, in the context of an artificial endometrial preparation cycle for an ET. This issue will be addressed using a large database of 7390 cycles performed in the past 5 years in IVI RMA Valencia (Spain), including both own and oocyte donation cycles. Results from this study could help to elucidate which is the optimal length of oestrogen exposure in order to maximize success rates in this type of ET cycles.

## 2 Materials and methods

### 2.1 Design and setting

Retrospective study conducted at IVI RMA Valencia (Spain) between November 2018 and July 2022. This study was approved by the Institutional Review Board of IVI RMA Valencia (Code: 2212-VLC-181-EL).

#### 2.1.1 Study population

The study enrolled 7390 infertile patients scheduled for an ET in the context of an artificial endometrial preparation cycle with HRT. Participating women were ≤ 50 years old with adequate endometrial pattern (triple layer) and thickness (6.5 mm) after estrogen treatment in the proliferative phase. Luteal phase support (LPS) was performed with micronized vaginal progesterone (MVP; 400 mg twice daily for 5 days) before ET. One or two blastocysts were transferred. Patients with uterine or adnexal anomalies were excluded from the study.

#### 2.1.2 Endpoints

The primary endpoint was the ongoing pregnancy rate (OPR) based on the length of oestrogen exposure before the onset of exogenous progesterone administration, in the overall population and according to the type of cycle (oocyte donation, own oocytes and own oocytes with PGT-A). OPR was defined as the presence of at least one viable fetus beyond Week 12.

Secondary endpoints included: the comparison of the mean length of oestrogen exposure according to pregnancy outcome variables (biochemical pregnancy, clinical pregnancy, biochemical miscarriage and clinical miscarriage), in the overall population and according to the type of cycle (oocyte donation, own oocytes without PGT-A, own oocytes with PGT-A); the impact of the length of oestrogen exposure on biochemical, clinical and ongoing pregnancy rates taking into account confounding variables (such as embryo quality, serum progesterone (P4) levels on the ET day, age, BMI, route of oestrogen administration, etc.); the assessment of pregnancy outcomes according to the length of oestrogen exposure as a categorical variable (divided into 4 different groups taking into account quartiles), in the overall population and according to the type of cycle.

Pregnancy outcome was determined by a positive β-hCG test (serum levels of β-hCG > 10 IU/ml 11 days after ET); clinical pregnancy was defined as the presence of at least one gestational sac on ultrasound; implantation was defined as the presence of a gestational sac per embryo transferred; miscarriage rate was defined as any pregnancy loss before Week 12, including biochemical miscarriage with a positive β-hCG test without evidence of a gestational sac and clinical miscarriage after confirmation of an intrauterine gestational sac; ectopic pregnancy was defined as a gestational sac located outside the uterine cavity; and LBR was defined as the number of deliveries that resulted in at least one live born neonate.

### 2.2 Study protocol

#### 2.2.1 Endometrial preparation

After transvaginal ultrasound to confirm ovarian quiescence, oestrogen treatment commenced on days 2–3 of menstruation. Oestrogens were administered orally at either 6 mg/day of estradiol valerate (Progynova®, Bayer Hispania, Barcelona, Spain; Meriestra®, Novartis, Barcelona, Spain) or transdermally with two patches of 75 mg estradiol hemihydrate (Evopad®, Janssen Cilag, Madrid, Spain) every 48 h. Patients who underwent egg donation cycles using fresh embryos were given a GnRH agonist (Decapepty® 3.75 mg, single dose, Ipsen Pharma, Barcelona, Spain) administered in the mid-luteal phase of the previous menstrual cycle, or a gonadotropin-releasing hormone (GnRH) antagonist (0.25 mg/day) for 5 days from the first day of menstruation (Orgalutran® 0.25 mg/0.5 ml, single dose, Merck Sharp & Dohme, Madrid, Spain) (10). After 10–14 days on estrogens, a vaginal two-dimensional (2D) ultrasound was performed to measure endometrial thickness (EMT) and to confirm a triple-layer pattern, and a blood sample was drawn for estradiol (E2) and P4 determinations to ensure that no spontaneous ovulation had occurred. If EMT was >6.5 mm, the endometrial pattern was trilaminar, and serum P < 1.0 ng/ml, ET was scheduled.

LPS began 5 days before ET with MVP at a dose of 400 mg twice daily (Utrogestan®, SEID, Barcelona, Spain; Progeffik®, Effik, Madrid, Spain; or Cyclogest®, Gedeon Richter, Barcelona, Spain). If pregnancy occurred, hormonal treatment was maintained until pregnancy week 12 in accordance with routine practice.

#### 2.2.2 IVF laboratory

Intracytoplasmic sperm injection (ICSI) was used in all cases, as it is the main method of fertilization used in our center. Either fresh or vitrified oocytes were used in oocyte donation cycles since there are no differences in pregnancy rates between them (11, 12). Likewise, ET was performed with fresh or thawed blastocysts.

Embryo quality was classified according to the Spanish ASEBIR (*Asociación para el estudio de la biología de la reproducción*) classification (13). This classification allocates each blastocyst to a category from A to D based on the trophectoderm and the inner cell mass morphology, being A the best and D the worst quality. Only embryos graded A to C were transferred.

Embryo transfers were performed in lithotomy position by senior gynecologists under transabdominal ultrasound guidance with full bladder.

### 2.3 Statistical analysis

Statistical analysis was performed using IBM SPSS Statistics v25 software (SPSS Inc., Chicago, IL, USA). Continuous variables were expressed as mean ± standard deviation (SD), whereas categorical variables were expressed as percentages.

Categorical variables were compared with a Chi-squared test, and a ANOVA test was used to compare the continuous variables between the two or more groups. P-value < 0.05 was considered statistically significant.

To analyze the correlation between the length of oestrogen therapy and pregnancy outcome, a binary logistic regression analysis was performed. Variables that were correlated to pregnancy outcome in a univariable analysis were included in the model (age, BMI, embryo quality (A, B or C), type of cycle (oocyte donation, own oocytes and own oocytes with PGT-A), route of oestrogen administration (patches or pills), and serum P4 levels on ET day).

The length of oestrogen therapy was also compared as a categorical variable grouped as: i) 9 categories according to percentiles (<10; 10<11; 11<12; 12<13; 13<14; 14<15; 15<17; 17 ≤ 19; >19 days), and ii) 2 categories according to the cut-off point of 14 days.

## 3 Results

### 3.1 Descriptive analysis

Of the 7390 HRT-ET cycles included in the study, 5044 (68.3%) involved oocyte donation cycles, 1049 (14.2%) were cycles with own oocytes and 1297 (17.6%) were cycles with own oocytes with PGT-A.

Cycles with own oocytes, with or without PGT-A, were always transferred in the context of a frozen embryo transfer (n=2346), while cycles with donated oocytes could be transferred in the context of a fresh embryo transfer (n=2066) or a frozen embryo transfer (n=2978).

Mean characteristics of the study population are shown in **Table 1**.

**Table 1:** Cycle and patients’ characteristics of the cycles analyzed.

	Overall N=7390	Oocyte donation N=5044	Own oocytes N=1049	Own oocytes with PGT-A N=1297	P value
Age (y.o.)	40.5 ± 4.8	42.1 ± 4.3	35.2 ± 3.8	38.3 ± 3.5	**<0.001**
BMI (kg/m^2^)	23.6 ± 4.2	23.7 ± 4.2	23.1 ± 4.2	23.3 ± 3.8	**<0.001**
Last serum E2 before progesterone onset (pg/mL)	312.7 ± 376.6	322.3 ± 387.0	290.0 ± 360.2	291.0 ± 344.2	**0.017**
Last serum P4 before progesterone onset (ng/mL)	0.21 ± 0.29	0.23 ± 0.27	0.18 ± 0.27	0.18 ± 0.37	**<0.001**
Last EMT before progesterone onset (mm)	8.9 ± 1.6	8.9 ± 1.7	9.0 ± 1.6	8.8 ± 1.5	**0.029**
Days oestrogen exposure until progesterone onset	14.2 ± 3.7	14.7 ± 3.8	12.8 ± 3.0	13.1 ± 3.1	**<0.001**
Serum P4 levels on day of ET (ng/mL)	13.2 ± 6.1	13.2 ± 6.1	12.8 ± 5.4	13.3 ± 6.6	0.070

E2, estradiol; P4, progesterone; EMT, endometrial thickness; ET, embryo transfer; PGT-A, preimplantational genetic testing for aneuploidies. Data are shown as mean ± standard deviation. ANOVA test. P-value < 0.05 is considered statistically significant.

Bold values, statistically significant differences.

Coloured line, is the variable in which we have focused in this article.

The range of oestrogen exposure prior to luteal phase supplementation ranged from 6 to 36 days in the overall population. In oocyte donation cycles it ranged from 6 to 36 days, from 6 to 28 days in cycles of own oocytes without PGT-A, and from 6 to 31 days in cycles with own oocytes with PGT-A.

Oestrogens were administered orally in the form of pills in 79.2% of cases, in the form of transdermal patches in 15.3% and using a combination of both in the remaining 5.5%.

Embryos transferred were classified as A in 18.9% of cases, as B in 65.0% of cases and as C in the remaining 16.1% of cases. The majority of cycles were single embryo transfers (92.2%), while 7.8% of them were double embryo transfers.

### 3.2 Clinical outcome according to the length of oestrogen exposure before progesterone onset

Mean ongoing pregnancy rate was 45.9%. Mean length of oestrogen exposure before progesterone administration onset was 14.3 ± 3.7 days in ongoing pregnancies vs. 14.1 ± 3.6 in non-ongoing pregnancies (p=0.044). This difference continued to be significant only in the subgroup of patients in oocyte donation cycles (14.9 ± 3.8 in ongoing pregnancies vs. 14.6 ± 3.7 in non-ongoing pregnancies; p=0.013) (**Table 2**) after the stratification of patients according to the type of cycle (oocyte donation, own oocytes and own oocytes with PGT-A).

**Table 2:** Mean length of oestrogen exposure (days) according to clinical outcome.

		Overall N=7390	Oocyte donation N=5044	Own oocytes N=1049	Own oocytes with PGT-A N=1297
Biochemical pregnancy (%)	Yes	14.19 ± 3.7	14.78 ± 3.8	12.79 ± 2.9	13.04 ± 3.0
	No	14.10 ± 3.6	14.60 ± 3.8	12.71 ± 3.0	13.29 ± 3.1
	P-value	0.274	0.102	0.645	0.148
Clinical pregnancy (%)	Yes	14.20 ± 3.7	14.80 ± 3.8	12.75 ± 2.9	13.02 ± 3.0
	No	14.11 ± 3.6	14.61 ± 3.7	12.77 ± 3.1	13.26 ± 3.2
	P-value	0.254	0.071	0.902	0.155
Ongoing pregnancy (%)	Yes	14.25 ± 3.6	14.86 ± 3.8	12.76 ± 3.0	13.05 ± 3.0
	No	14.08 ± 3.6	14.59 ± 3.7	12.76 ± 3.0	13.21 ± 3.1
	P-value	**0.044**	**0.013**	0.976	0.360
Biochemical miscarriage (%)	Yes	14.17 ± 3.6	14.65 ± 3.7	13.00 ± 3.0	13.25 ± 3.5
	No	14.16 ± 3.7	14.72 ± 3.8	12.74 ± 3.0	13.12 ± 3.0
	P-value	0.954	0.753	0.429	0.705
Clinical miscarriage (%)	Yes	13.90 ± 3.4	14.43 ± 3.6	12.72 ± 2.5	12.81 ± 2.8
	No	14.18 ± 3.7	14.74 ± 3.8	12.76 ± 3.0	13.16 ± 3.1
	P-value	0.051	0.086	0.880	0.247

Data are shown as mean ± standard deviation. Results are shown in the overall population and each type of cycle. ANOVA test. P-value < 0.05 is considered statistically significant.

Bold values, statistically significant p-value.

Also in oocyte donation cycles, the mean length of oestrogen exposure was significantly shorter in cycles that ended in a clinical miscarriage. In contrast, cycles with own oocytes with or without PGT-A didn’t show any significant differences in the length of oestrogen exposure according to pregnancy outcome (**Table 2**).

**Figure 1:**
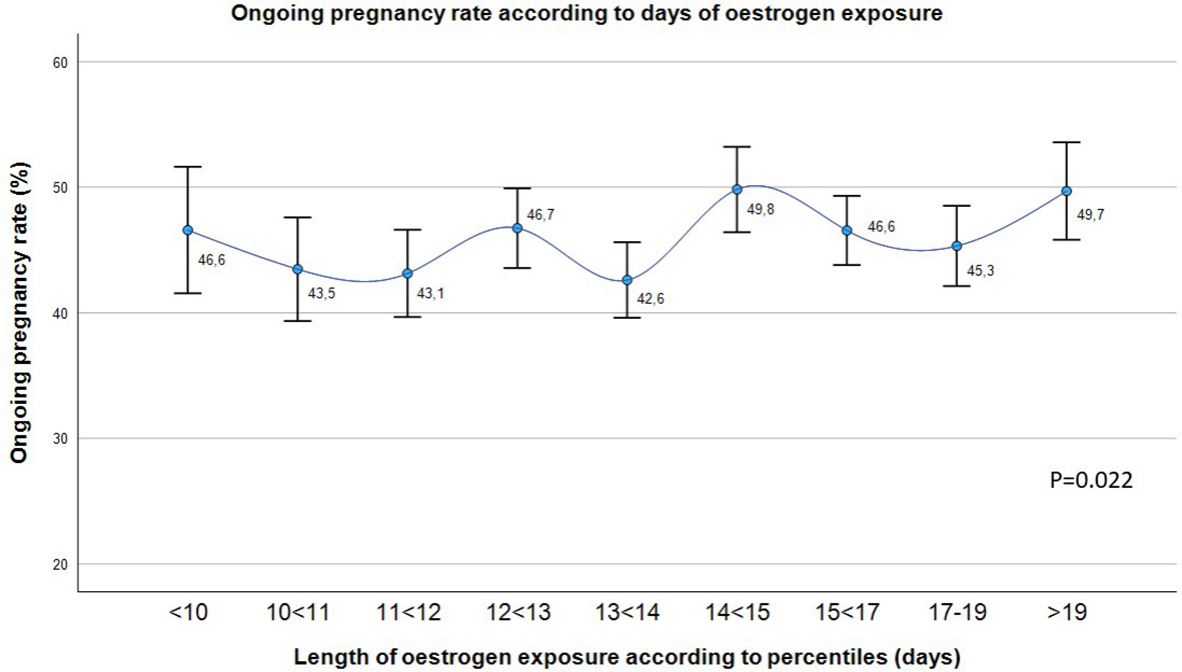
Ongoing pregnancy rate (%) in the overall population according to the length of oestrogen exposure in days divided into 9 groups. Chi-squared test. P-value < 0.05 is considered statistically significant.

OPR was statistically different according to the percentiles of oestrogen duration (p=0.022), although any trend was observed (**Figure 1**).

When classifying the population into 2 groups according to be above or below the cut-off point of 14 days of oestrogen exposure, a higher miscarriage rate was observed in patients with a shorter duration of oestrogens (18.3 vs 15.8%, p=0.040). **Table 3** shows the clinical outcomes according to the length of oestrogen exposure (≤ or > 14 days), in the overall population and in the three types of cycle.

**Table 3:** Clinical outcome according to the length of oestrogen exposure as a categorical variable, divided into 2 groups according to the cut-off of 14 days.

	Overall N=7390			Oocyte donation N=5044			Own oocytes N=1049			Own oocytes with PGT-A N=1297		
	≤ 14	> 14	p-value	≤ 14	> 14	p-value	≤ 14	> 14	p-value	≤ 14	> 14	p-value
Biochemical pregnancy (%)	61.7	62.2	0.654	61.3	62.8	0.278	61.8	62.1	0.921	62.6	58.1	0.142
Clinical pregnancy (%)	53.8	54.4	0.598	53.4	55.3	0.190	53.1	52.7	0.907	55.5	50.1	0.087
Ongoing pregnancy (%)	45.3	46.9	0.199	44.9	47.7	**0.046**	43.5	44.0	0.894	48.0	43.1	0.117
Biochemical miscarriage (%)	11.9	12.0	0.947	12.2	11.5	0.571	13.3	13.9	0.836	10.0	13.7	0.148
Clinical miscarriage (%)	18.3	15.8	**0.040**	18.8	15.4	**0.021**	20.1	18.8	0.738	15.4	15.9	0.883

Results are shown in the overall population and each type of cycle. Chi-squared test. P-value < 0.05 is considered statistically significant.

Bold values, statistically significant p-value.

Logistic regression model showed that the length of oestrogen exposure was not an independent factor for increasing the rate of biochemical pregnancy (OR: 1.00 IC95% (0.99-1.01), p=0.99), clinical pregnancy (OR: 1.00 IC95% (0.99-1.01), p=0.91) and ongoing pregnancy (OR: 1.01 IC95% (0.99-1.02), p=0.38), after adjusting for all confounding variables mentioned above.

## 4 Discussion

This retrospective analysis of more than 7000 HRT cycles for embryo transfer suggests that the length of oestrogen exposure (in days) before exogenous progesterone administration do not affect clinical outcomes.

Despite ongoing pregnancies had a significantly longer duration of oestrogen exposure, the difference with respect to non-ongoing pregnancies is less than one day (14.3 ± 3.7 vs. 14.1 ± 3.6; p=0.044), thus not clinically relevant. This finding was observed in the overall population and particularly in oocyte donation cycles (**Table 2**).

However, the subsequent division of the length of oestrogen exposure into 9 groups according to percentiles shows how this statistically significant variation in OPR is not clinically relevant (the maximum difference between 2 groups is of 7 points) (**Figure 1**). Hence, statistically significant differences may be due to the large sample size analyzed.

In particular, the maximum difference in OPR happens around day 14 of oestrogen exposure (**Figure 1**), so that this was the cut-off point chosen to study the behavior of pregnancy rates. In addition, the mean number of days of oestrogen exposure is significantly different between the different types of cycles (oocyte donation, own oocytes and own oocytes with PGT-A) (**Table 1**), probably due to the necessity of synchronization between the donor and the recipient in oocyte donation cycles. For this reason, and also in order to avoid any potential bias related to embryo quality, its impact on pregnancy outcome has been also addressed separately.

Despite a slight tendency to higher pregnancy rates in cycles with longer duration of oestrogen therapy (lower clinical miscarriage rates and higher ongoing pregnancy rates in oocyte donation cycles, although only lower clinical miscarriage rates in the overall population when >14 days), these differences were also not clinically relevant (**Table 3**).

This slight tendency to better results with longer oestrogen exposure contrasts with previous studies found in the literature, which have claimed that shorter periods of oestrogen exposure are more beneficial to pregnancy outcome [less than 24 days until progesterone onset in Bourdon et al. (4) and less than 20 days in Sunkara et al. (6)]. In addition, Borini and Younis proposed the optimal length of oestrogen exposure in oocyte donation cycles to be between 12 and 19 days (7, 8). In contrast, we didn’t see any significant drop in pregnancy rates beyond 19 days (**Table 3**), also in line with previously evidences proving that periods of oestrogen exposure longer than 35 days in oocyte donation cycles do not hamper clinical outcomes (14).

Regarding cycles with own oocytes, our results don’t show any difference in pregnancy outcomes regarding the length of oestrogen exposure, both with or without PGT-A (**Table 3**), as previously evidenced (2, 3). The number of days covered by these two studies ranges from 5 to 34, very similar to the range of the present study (6 to 36 days).

Hence, in this study we were not able to propose a potential optimal window for the length of oestrogen exposure in HRT-ET cycles, as the statistically significant differences observed are not clinically relevant. Indeed, binary regression models demonstrated that the length of oestrogen exposure was not an independent factor for increasing the rate of biochemical pregnancy, clinical pregnancy and ongoing pregnancy, after adjusting for all confounding variables (including the type of cycle).

The main advantage of HRT cycles for an ET is the flexibility they offer, allowing the best adjustment of dates taking into account the patient, the doctor and the clinic needs. This flexibility is possible by modifying the duration of oestrogen exposure until the onset of exogenous progesterone administration. Results from this study suggest that the variation in the length of oestrogen therapy do not exert any impact on pregnancy outcome, offering the possibility to continue with this clinical practice and favoring the logistics of this type of cycles to suit individual needs. Nevertheless, despite the large sample size analyzed, the main limitation of this study is its retrospective design and conclusions should be taken with caution.

## Data availability statement

The raw data supporting the conclusions of this article will be made available by the authors, without undue reservation.

## Ethics statement

The studies involving human participants were reviewed and approved by Research Ethics Committee of IVI Valencia. Written informed consent for participation was not required for this study in accordance with the national legislation and the institutional requirements. Written informed consent was not obtained from the individual(s) for the publication of any potentially identifiable images or data included in this article.

## Author contributions

EL and CR-V significantly contributed to the study conception and design. MS-M significantly contributed to the preparation of the database used for the subsequent analysis included in this study, along with CR-V. CR-V performed statistical analyses and data interpretation, and drafted the article. MS-M also contributed to the elaboration of the background and context of the drafted article. EL carefully revised the drafted article. All authors contributed to the article and approved the submitted version.
